# Altered gut microbiota in the early stage of acute pancreatitis were related to the occurrence of acute respiratory distress syndrome

**DOI:** 10.3389/fcimb.2023.1127369

**Published:** 2023-03-06

**Authors:** Xiaomin Hu, Ziying Han, Ruilin Zhou, Wan Su, Liang Gong, Zihan Yang, Xiao Song, Shuyang Zhang, Huijun Shu, Dong Wu

**Affiliations:** ^1^ Department of Medical Research Center, State Key Laboratory of Complex Severe and Rare Diseases, Peking Union Medical College Hospital, Chinese Academy of Medical Sciences and Peking Union Medical College, Beijing, China; ^2^ Department of Gastroenterology, State Key Laboratory of Complex Severe and Rare Diseases, Peking Union Medical College Hospital, Chinese Academy of Medical Sciences and Peking Union Medical College, Beijing, China; ^3^ Department of Cardiology, Peking Union Medical College Hospital, Chinese Academy of Medical Science and Peking Union Medical College, Beijing, China; ^4^ Department of Endocrinology, Peking Union Medical College Hospital, Peking Union Medical College, Chinese Academy of Medical Sciences, Beijing, China; ^5^ Department of Emergency Medicine, Peking Union Medical College Hospital, Peking Union Medical College, Chinese Academy of Medical Sciences, Beijing, China

**Keywords:** acute pancreatitis, acute respiratory distress syndrome, gut microbiota, disease prediction, biomarker

## Abstract

**Background:**

Acute respiratory distress syndrome (ARDS) is the most common cause of organ failure in acute pancreatitis (AP) patients, which associated with high mortality. Specific changes in the gut microbiota have been shown to influence progression of acute pancreatitis. We aimed to determine whether early alterations in the gut microbiota is related to and could predict ARDS occurrence in AP patients.

**Methods:**

In this study, we performed 16S rRNA sequencing analysis in 65 AP patients and 20 healthy volunteers. The AP patients were further divided into two groups: 26 AP-ARDS patients and 39 AP-nonARDS patients based on ARDS occurrence during hospitalization.

**Results:**

Our results showed that the AP-ARDS patients exhibited specific changes in gut microbiota composition and function as compared to subjects of AP-nonARDS group. Higher abundances of Proteobacteria phylum, *Enterobacteriaceae* family, *Escherichia-Shigella* genus, and *Klebsiella pneumoniae*, but lower abundances of *Bifidobacterium* genus were found in AP-ARDS group compared with AP-nonARDS groups. Random forest modelling analysis revealed that the *Escherichia-shigella* genus was effective to distinguish AP-ARDS from AP-nonARDS, which could predict ARDS occurrence in AP patients.

**Conclusions:**

Our study revealed that alterations of gut microbiota in AP patients on admission were associated with ARDS occurrence after hospitalization, indicating a potential predictive and pathogenic role of gut microbiota in the development of ARDS in AP patients.

## Introduction

Acute pancreatitis (AP), one of the most common gastrointestinal diseases, is an acute inflammatory disease with an increasing incidence worldwide (Tenner et al., 2013; Greenberg et al., 2016). In patients with AP, persistent organ failure (OF) can reach 35% and is a key determinant of mortality (Johnson and Abu-Hilal, 2004; Shah and Rana, 2020). The most common cause of OF in AP is acute respiratory distress syndrome (ARDS) (Garg et al., 2005). ARDS is a type of acute, diffuse inflammatory lung injury that can lead to a high mortality rate of up to 48% (Schmandt et al., 2021). Even after five years of rehabilitation, surviving ARDS patients still suffer from poor long-term quality of life; including exercise limitation, difficulty in returning to work, and high medical costs (Herridge et al., 2011). However, missed or delayed diagnosis of ARDS remains a common and challenging problem worldwide. Nearly two-thirds of patients had a delayed or missed diagnosis of ARDS. The miss rate was approximately 40%, and the diagnosis of half of mild ARDS patients was delayed (Bellani et al., 2020). Early recognition of ARDS ensures that patients receive appropriate treatment which relieves lung injury and improves prognosis, therefore, effective prediction methods for ARDS are urgently required (Fan et al., 2018; Pan et al., 2018; Bellani et al., 2020).

AP is strongly associated with gut microbiota imbalance and an impaired epithelial barrier (Besselink et al., 2009). Compared with the healthy group, the diversity of the gut microbiota decreased; with a greater abundance of pathogenic bacteria in AP patients and lower numbers of commensal beneficial genera (Zhang et al., 2018; Zhu et al., 2019). According to the revised Atlanta classification 2012, AP can be divided into three grades: mild AP (MAP), moderately severe AP (MSAP) with transient OF, and severe AP (SAP) with persistent OF (Banks et al., 2013). In AP patients with different degrees of severity, the dominant gut microbiota also varied; with *Bacteroides* in MAP, *Escherichia-Shigella* in MSAP, and Enterococcus in SAP (Yu et al., 2020). Similar results have been reported in animal models; gut microbiota-depleted AP rats were found to have lower levels of inflammatory factors (Zheng et al., 2019; Li et al., 2020). The degree of gut barrier injury and bacterial translocation are important prognostic factors for AP (Besselink et al., 2009).

Disorganized microbiota and damaged intestinal epithelium in AP patients make it easier for the endotoxin diffusion, immune cell migration, and bacteria translocation. The lung environment may be more susceptible to the gut microbiota in patients with AP. Owing to the increased gut permeability, inflammatory factors and activated trypsin could function as the gut-lung axis, thus triggering and promoting lung disease in patients with AP (Shah and Rana, 2020). In addition, gram-negative infections promote release of endotoxins and these can translocate through high-permeability gut mucosa and contribute to the development of ARDS in AP patients (AP-ARDS; “ARDS” mean for ARDS in general, “AP-ARDS” mean for ARDS in acute pancreatitis.) (Gray et al., 2003). Bacteria can also translocate from the gut to the lung (Mukherjee and Hanidziar, 2018). Previous studies also found evidence of bacteria translocation in ARDS patients (Dickson et al., 2016; Dickson et al., 2020). The composition of gut-associated bacteria, especially *Bacteroidetes* and *Enterobacteriaceae*, increased in the lower respiratory tract of ARDS patients (Dickson et al., 2016; Siwicka-Gieroba and Czarko-Wicha, 2020). Further study found that the increase of Escherichia coli in lung was related to higher mortality of ARDS patients (Zhang et al., 2021). Studies have confirmed that the lung microbiota is associated with alveolar inflammation in ARDS (Dickson et al., 2016). Michihito et al. revealed that alterations in lung microbiota are correlated with serum IL-6 levels and hospital mortality in patients with ARDS (Kyo et al., 2019). Therefore, the gut microbiota may be involved in the pathogenesis of AP-ARDS, however, the relationship between the gut microbiota and AP-ARDS remains unknown. If early changes in gut microbiota in AP-ARDS patients can be found, they may help in the early recognition of AP-ARDS, promote early intervention, and even improve patient outcomes.

Therefore, we wanted to investigate the relationship between gut microbiota and AP-ARDS by comparing the microbiota among three groups: healthy controls, AP patients without ARDS (AP-nonARDS), and AP-ARDS patients. By collecting the gut microbiota at the early stage of AP, we investigated whether gut microbiota was related to and could help predict and recognize AP-ARDS. Our study explored the potential effect of the gut-lung axis in AP-ARDS and provide identify biomarkers for prediction and early recognition of AP-ARDS.

## Methods

### Study population

This prospective and observational cohort study was conducted at Peking Union Medical College Hospital, Beijing, China. Twenty healthy volunteers and 75 patients were enrolled between June 2018 and July 2021. Ten AP patients were excluded due to history of comorbidities and medicine intake. All patients fulfilled the AP diagnostic criteria according to the 2012 revised Atlanta criteria and were admitted within 24 h of onset (Banks et al., 2013).

The exclusion criteria were as follows: patients with chronic pancreatitis, immunosuppressive disease, inflammatory bowel disease, cancer, irritable bowel syndrome, gastroenteritis, or necrotizing enterocolitis; and use of antibiotics, probiotics, laxatives, or Chinese herbs within two months before symptom onset. Informed consent was obtained from all participants. This study was approved by the Ethics Committee of PUMCH (Identifier: JS1826; date of approval:20th February 2018. Period of validity: February 2018 to August 2020)

For all patients, no ARDS was diagnosed during the first fecal sampling; however, some patients developed ARDS during hospitalization. Patients were diagnosed with ARDS according to the Berlin definition (Ranieri et al., 2012). According to PaO2/FiO2 levels, patients with ARDS were divided into three groups: mild ARDS (MARDS), moderate ARDS, and severe ARDS (Ranieri et al., 2012). Considering the higher rate of mechanical ventilation in moderate ARDS and severe ARDS patients (Fan et al., 2018) as well as the small sample size of severe ARDS group (n=5), we combined moderate ARDS and severe ARDS as non-MARDS group for subgroup analysis.

### Collection and analysis of clinical characteristics

Demographic and clinical data were collected from medical record libraries, including age, sex, body mass index (BMI), smoking history, drinking history, combined diseases, disease severity-related scores, local complications, systematic complications, and clinical outcomes. Definitions of local and systematic complications can be found in previous studies (Yu et al., 2020; Hu et al., 2021b; Yu et al., 2021).

Statistical analysis of clinical characteristics was performed using SPSS Statistics 26.0 (IBM Corp., Armonk, NY, USA). The mean ± standard deviation (SD) was used to represent the data distribution. However, when the data did not fit a normal distribution, the median (interquartile range [IQR]) was used. For categorical variables, we performed the χ2 test or Fisher’s exact test; while for continuous variables, we performed the nonparametric Mann-Whitney test. A difference was considered significant when the two-sided p value was less than 0.05.

### Sample collection, DNA extraction, and 16S rRNA gene sequencing

Patients with AP have difficulty defecating owing to fasting and water deprivation. Therefore, we used rectal swabs for fecal sampling, as previous studies have described (Yu et al., 2020; Yu et al., 2021). The fecal samples were immediately collected after admission, and all samples were collected within 24 h of AP onset. Then, these samples were stored at − 80°C, and microbial DNA was extracted as soon as possible. We then performed PCR amplification, library construction, Illumina (San Diego, CA, USA) MiSeq sequencing, and sequence quality control, using previously reported methods (Yu et al., 2020; Yu et al., 2021).

### Bioinformatics analysis

Amplicon sequence variant (ASV) analysis was performed using EasyAmplicon (Version 1.10). We use the -derep_fullength command in VSEARCH (version 2.15) to create dereplication, denoised these unique sequences into ASVs by the -unoise3 algorithm in USEARCH (Version 10.0), created an ASVs table using the -usearch_global command, and then completed the ASVs classification using the Sintax algorithm command.

### Microbiota composition

Alpha diversity analysis, including the Chao and Simpson indices, was performed using Mothur software (1.30.2). The dilution curve was plotted using R software to calculate the microbial diversity at different numbers of sequences. In the beta diversity analysis, principal coordinate analysis (PCoA) was performed using the R package vegan (v2.5-6).

Based on taxonomic information, community structure analysis can be performed at various taxonomic levels. The composition of microbiota at the phylum, family, genus, and species levels was determined using the stat package in R software. Relevant analytical methods were used to detect variation in microbes between the different groups and pairwise comparisons were calculated using the Wilcoxon rank-sum test.

### Functional annotation

Linear discriminant analysis (LDA) effect size (LefSe; http://huttenhower.sph.harvard.edu/galaxy) was performed to identify potential biomarkers in the different groups (LDA score>2, p<0.05). Microbiota phenotypes were predicted using BugBase, based on normalized ASVs. The significance of the functional difference was evaluated using the Wilcoxon rank-sum test in the BugBase prediction analysis. The Random Forest R package was used to build a random forest regression model. We randomly divided the 65 samples into training sets (70%) and testing sets (30%) according to the 16S amplicon sequence and clinical characteristics. Bioinformatics analysis and visualization were performed using the R software. Detailed analysis methods can be found in our previous studies (Hu et al., 2021a; Hu et al., 2021b).

## Results

### Clinical characteristics of AP-ARDS patients

Sixty-five AP patients and 20 healthy individuals were included in the study. Rectal swabs were collected before the occurrence of ARDS. Twenty-six patients with AP developed ARDS (AP-ARDS; mild ARDS: n=12; moderate ARDS: n=9; severe ARDS: n=5) and 39 patients did not (AP-nonARDS). The average diagnosis time of ARDS was 3.46 ± 1.92 d after AP onset. **Table 1** shows the demographic and clinical characteristics of the two groups. Demographic characteristics were generally balanced, however, patients with ARDS had more severe symptoms than those without ARDS (**Table 1**). The AP-ARDS group had a higher proportion of SAP (2.56% vs. 73.08%; *p*<0.001) and higher disease severity-related scores compared to the AP-nonARDS group. The occurrence of acute peripancreatic fluid collection (35.90% vs. 92.31%; *p*<0.001), systematic complications, organ failure (10.26% vs. 100.00%; *p*<0.001), and ICU admission (0.00% vs. 73.08%; *p*<0.001) was also significantly increased in the AP-ARDS group, except for bowel obstruction and mental status. Furthermore, the total duration of organ failure (median 0.00, IQR 0.00–0.00; vs median 85.00, IQR 41.50–276.00; *p*<0.001); ICU stay (0.00 ± 0.00 vs. 7.15 ± 6.59; *p*<0.001) and hospital stay (8.26 ± 6.65 vs. 23.04 ± 11.52; *p*<0.001) were both longer in AP-ARDS group.

**Table 1:** Demographic and clinical characteristics of health control, AP-nonARDS, and AP-ARDS group.

	CONTROL (n=20)	AP-nonARDS (n=39)	AP-ARDS (n=26)	P value (AP-nonARDS vs AP-ARDS)
Age (years), mean ± SD	37.20 ± 12.00	44.15 ± 15.02	48.69 ± 13.99	0.135
Male, n (%)	11(55.00)	17(43.59)	17(65.38)	0.085
BMI (kg/m2), mean ± SD	22.80 ± 2.89	26.19 ± 3.63	26.48 ± 3.88	0.794
Smoking, n (%)		9(23.07)	9(34.62)	0.308
Drinking, n (%)		9(23.07)	7(26.92)	0.724
Comorbid abnormalities, n (%)				
Hypertension		10(25.64)	13(50.00)	0.044
Diabetes		9 (23.07)	8(30.77)	0.489
Fatty liver		27(69.23)	16(61.54)	0.521
Etiology, n (%)				0.538
Biliary		18(46.15)	9(34.62)	
Hypertriglyceridemia		17(43.59)	15(57.69)	
Alcohol consumption		4(10.26)	2(7.69)	
Disease severity, n (%)				<0.001
MAP		21(53.85)	0(0.00)	
MSAP		17(43.59)	7(26.92)	
SAP		1(2.56)	19(73.08)	
APACHE II, mean ± SD		3.13 ± 2.25	9.96 ± 4.17	<0.001
SOFA score, mean ± SD; median (IQR)		0.54 ± 0.64; 0.00 (0.00,1.00)	6.12 ± 4.09; 4.00(3.00, 7.25)	<0.001
Balthazar score E, mean ± SD		2.90 ± 1.02	4.04 ± 0.72	<0.001
Local complications, n (%)				
Acute peripancreatic fluid collection (APFC)		14(35.90)	24(92.31)	<0.001
Pancreatic pseudocyst (PP)		3(7.69)	2(7.69)	>0.999
Acute necrotic collection (ANC)		2(5.13)	12(46.15)	<0.001
Walled off necrosis (WON)		0(0.00)	2(7.69)	0.079
Infected necrosis		0(0.00)	8(30.77)	0.001
Systematic complication, n (%)				
Systemic inflammatory response syndrome (SIRS)		13(33.33)	22(84.62)	<0.001
Acute kidney injury		1(2.56)	12(46.15)	<0.001
Shock		0(0.00)	10(38.46)	<0.001
Liver damage		1(2.56)	11(42.31)	<0.001
Myocardial injury		1(2.56)	6(23.08)	0.009
Sepsis		1(2.56)	14(53.85)	<0.001
Abdominal compartment syndrome (ACS)		1(2.56)	7(26.92)	0.003
Bowel obstruction		3(7.69)	7(26.92)	0.035
Outcome				
Organ failure, n (%)		4(10.26)	26 (100.0)	<0.001
Organ failure duration (h), mean ± SD; median (IQR)		2.59 ± 8.30; 0.00(0.00,0.00)	164.65 ± 159.85; 85.00(41.50,276.00)	<0.001
ICU, n (%)		0(0.00)	19(73.08)	<0.001
ICU stay (days), mean ± SD;		0.00 ± 0.00	7.15 ± 6.59	<0.001
Hospital stay (days), mean ± SD		8.26 ± 6.65	23.04 ± 11.52	<0.001
Death, n (%)		0(0.00)	1(3.85)	0.217

### Taxonomic features of gut microbiota in AP-ARDS patients

**Figure 1:**
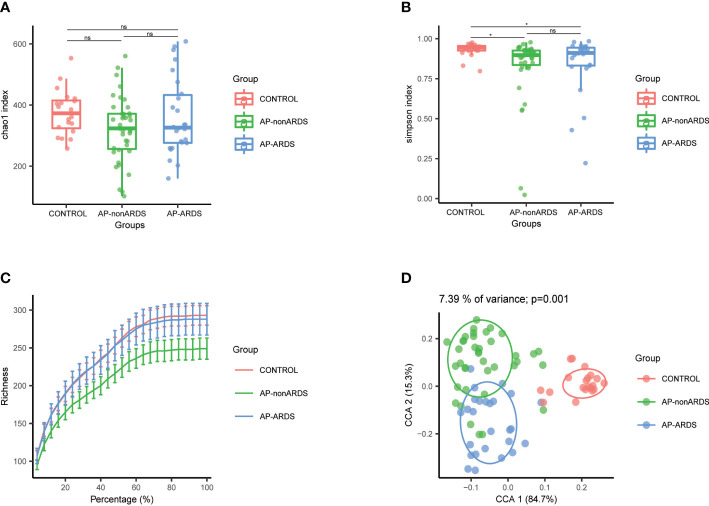
Diversity analysis of Control, AP-nonARDS and AP-ARDS group. **(A)** Chao index of α analysis; **(B)** Simpson index of α analysis. There are significant differences between the Control group and AP patients, but no significant difference between AP-nonARDS and AP-ARDS group. **(C)** Rarefaction curves analysis. **(D)**. Principal coordinate analysis (PCoA). CONTROL, healthy population; AP-nonARDS, AP patients without ARDS; AP-ARDS, AP patients with ARDS. * P < 0.05; ns: not significant.

We analyzed 745,895 reads that were clustered into 1910 ASVs. No statistically significant differences in the richness and diversity of the gut microbiota were noted between the AP-nonARDS and AP-ARDS groups. In the alpha diversity analysis, there were no significant differences in the Chao index (*p*>0.05 between any two groups; **Figure 1A**). Compared with healthy controls, the Simpson index decreased in both the AP-nonARDS and AP-ARDS groups, but no differences were found between the AP-nonARDS and AP-ARDS groups (**Figure 1B**). In the rarefaction curve analysis, the curve tended to plateau as the number of reads increased, demonstrating that microbiota in the healthy control, AP-nonARDS, and AP-ARDS groups were abundant and evenly distributed (**Figure 1C**). PCoA for the beta diversity results clearly distinguished the three groups, but overlap did occur between the AP-nonARDS group and AP-ARDS group. This indicated a significant difference in the microbiota structure between healthy controls and patients with AP, possible similarities between the AP-nonARDS group and AP-ARDS group (**Figure 1D**).

**Figure 2:**
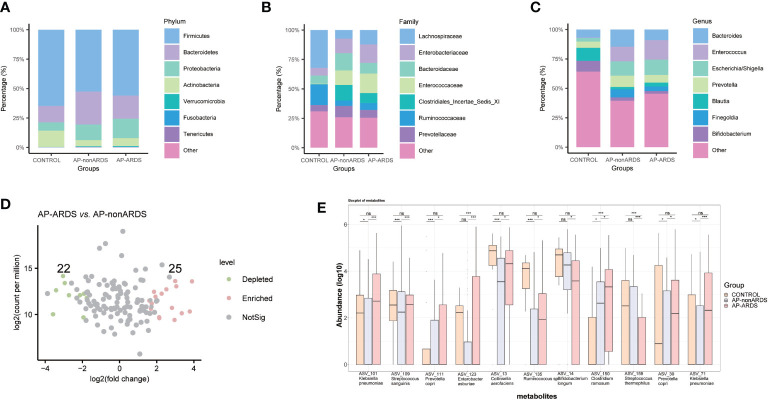
Gut microbiota composition at **(A)** phylum, **(B)** family, **(C)** genus levels. **(D)** Different amplicon sequence variants (ASVs) between AP-ARDS and AP-nonARDS group. (green= depleted in AP-ARDS group; red = enriched in AP-ARDS group; gray = no significantly difference). **(E)** Relative abundances of different species between AP-nonARDS and AP-ARDS groups. * P < 0.05; *** P < 0.001; ns: not significant.

The composition of the gut microbiota was significantly different among the three groups. At the phylum level, *Proteobacteria* and *Bacteroidetes* were both increased in patients with AP compared to healthy controls. *Proteobacteria* showed a gradually increase with disease progression (**Figure 2A**). At the family level, *Enterobacteriaceae*, *Enterococcaceae*, *Bacteroidaceae*, *Clostridiales Incertae Sedis XI*, and *Prevotellaceae* increased, while *Ruminococcaceae* decreased in patients with AP compared to healthy controls. In particular, the abundance of *Enterobacteriaceae* and *Enterococcaceae* increased with disease progression (**Figure 2B**). At the genus level, *Escherichia-Shigella*, *Bacteroides*, and *Enterococcus* were more abundant in patients with AP, while *Bifidobacterium* and *Blautia* were more abundant in healthy controls. *Escherichia-Shigella* and *Enterococcus* gradually increased while *Bifidobacterium* decreased with disease progression (**Figure 2C**). Compared to the AP-nonARDS group, 25 ASVs were enriched and 22 ASVs were depleted in the AP-ARDS group (**Figure 2D**). **Figure 2E** shows the top 11 different bacteria between the AP-ARDS and AP-nonARDS groups at the species level. *Klebsiella pneumoniae* (ASV_101, p<0.001; ASV_71, p<0.001), *Prevotella copri* (ASV_30, p<0.001; ASV_111, p=0.002), and *Clostridium ramosum* (ASV_150, p=0.002) showed a significant increase; and *Bifidobacterium longum* (ASV_14, p=0.003) decreased in the AP-ARDS group compared to the AP-nonARDS group. Among these microbiota, *Clostridium ramosum* (ASV_150) showed a gradual increase in the healthy to AP-nonARDS to AP-ARDS groups, whereas *Bifidobacterium longum* (ASV_40) showed a gradual decrease (**Figure 2E**).

**Figure 3:**
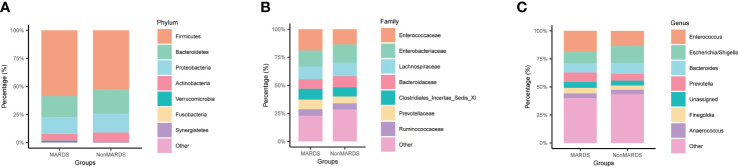
Subgroup analysis of gut microbiota composition at phylum **(A)**, family **(B)**, and genus **(C)** levels. MARDS, mild ARDS; NonMARDS, moderate ARDS and severe ARDS.

We performed a subgroup analysis according to ARDS severity, and identified some microbiota showing similar trends. At the phylum level, *Proteobacteria* increased in the non-MARDS group compared to that in the MARDS group (**Figure 3A**). At the family level, *Enterobacteriaceae* increased in the non-MARDS group (**Figure 3B**). At the genus level, *Escherichia-Shigella* was more abundant in the non-MARDS group (**Figure 3C**).

### Alterations of gut microbiota in AP-ARDS patients are associated with more severe manifestations

**Figure 4:**
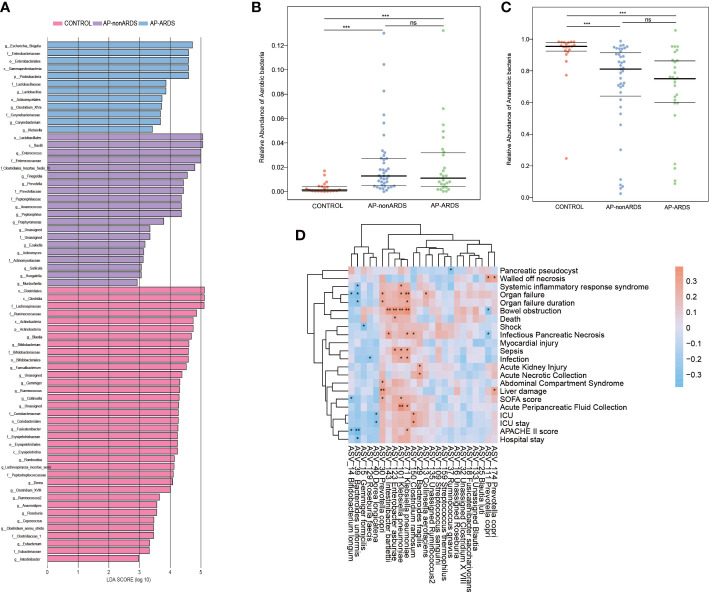
Microbial Function Analysis and Clinical Correlation Analysis. **(A)**. Linear discriminant analysis (LDA) Effect Size (LEfSe) analysis. **(B)**. Relative abundance of aerobic bacteria in BugBase analysis. **(C)**. Relative abundance of anaerobic bacteria in BugBase analysis. **(D)**. Spearman correlation of clinical characteristics and different species between AP-ARDS and AP-nonARDS group. * P < 0.05; *** P < 0.001, ns: not significant.

LEFSe analysis also revealed that *Enterobacteriaceae* and *Escherichia-Shigella* were dominant in AP-ARDS group while *Enterococcaceae* and *Enterococcus* were dominant in AP-nonARDS group (**Figure 4A**).

BugBase functional analysis predicted oxygen utilizing, gram staining, oxidative stress tolerance, biofilm forming, pathogenic potential, mobile element containing, and oxygen tolerance. Compared with healthy controls, anaerobic bacteria decreased in the AP-nonARDS and AP-ARDS groups (CONTROL vs. AP-nonARDS, CONTROL vs. AP-ARDS, both *p*<0.001). Although there was no significant difference, anaerobic bacteria showed a decreasing trend in AP-ARDS compared with AP-nonARDS (**Figure 4B**). In contrast, aerobic bacteria increased in the AP-nonARDS and AP-ARDS groups (**Figure 4C**).

Spearman correlation analysis was performed to investigate the relationship between microbiota and clinical outcomes. Two subspecies of *Klebsiella pneumoniae*, ASV_101 and ASV_71, were positively correlated with multiple clinical characteristics, including organ failure, bowel obstruction, sepsis, infection, and acute peripancreatic fluid collection. *Prevotella copri* (ASV_30) was positively correlated with the occurrence and duration of organ failure. Clostridium ramosum (ASV_150) was associated with ICU admission and length of hospital stay. As a probiotic, *Bifidobacterium longum* (ASV_14) negatively correlated with organ failure, Sequential Organ Failure Assessment score (SOFA score), and Acute Physiology And Chronic Health II score (APACHII score) (**Figure 4D**).

### The progression of AP-ARDS is closely associated with Enterobacteriaceae

**Figure 5:**
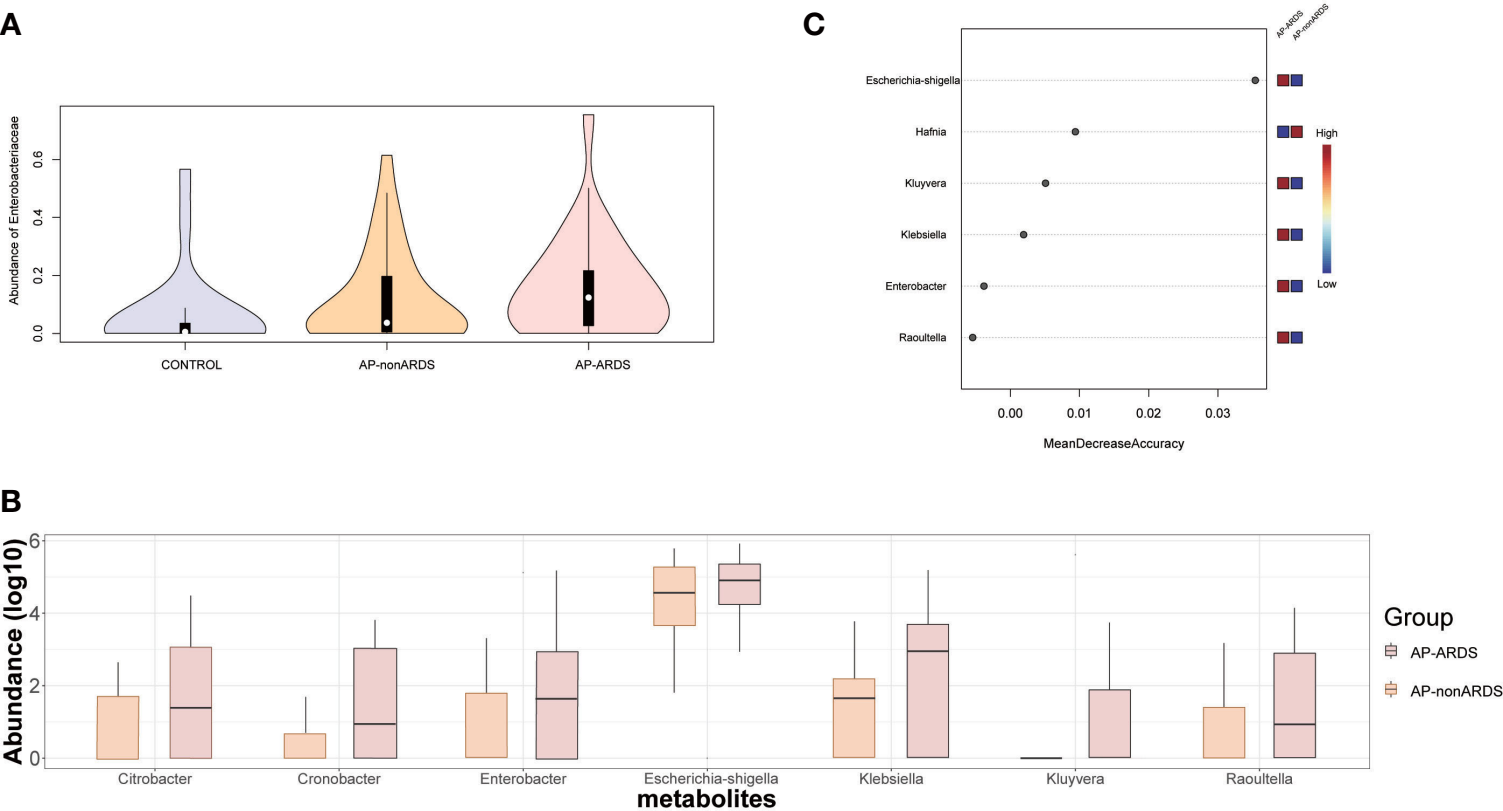
*Enterobacteriaceae* Analysis and Model Predicting. **(A)** The relative abundance of *Enterobacteriaceae* in Control, AP-nonARDS, and AP-ARDS group. **(B)** Major genus in *Enterobacteriaceae* family between AP-nonARDS, and AP-ARDS group. **(C)** Random forest model predicting. It screened out the *Escherichia-shigella* genus as the most significant feature for predicting ARDS.

Considering the significant increase in *Enterobacteriaceae* and its potential pathogenicity, we performed further analyses of *Enterobacteriaceae.* **Figure 5A** shows the relative abundance of *Enterobacteriaceae* increased with disease progression. Further analysis revealed that almost all genera of the *Enterobacteriaceae* family were increased in the AP-ARDS group (**Figure 5B**). Random forest identified *Escherichia-shigella* as the most significant feature for distinguishing AP-ARDS from AP-nonARDS (**Figure 5C**).

## Discussion

To our knowledge, this is the first study to explore the relationship between gut microbiota and AP-ARDS and reveals gut microbiota as a predictive biomarker for ARDS. The 16S rRNA sequencing analysis revealed differences of microbiota composition and function between the AP-ARDS and AP-nonARDS groups. Subgroup analysis suggested that gut microbiota composition was also related to the severity of ARDS. Before patients were diagnosed with AP-ARDS, the gut microbiota already had the characteristics of ARDS in AP patients. This indicates that the gut microbiota can be a potential biomarker for prediction and early recognition of AP-ARDS, thereby improving AP-ARDS diagnosis and treatment.

In our characteristics analysis, AP patients with ARDS were more serious than that in the non-ARDS group. AP-severity-associated changes in the gut microbiota were also observed in the AP-ARDS group compared with the AP-nonARDS group. However, we also observed some microbiota changes that might be related to the occurrence and development of AP-ARDS. The enrichment of *Enterobacteriaceae* and *Escherichia-Shigella*, and the reduction of *Bifidobacterium* were associated with AP-ARDS. Previous studies have focused on the lung microbiota of ARDS patients and found that the composition was affected by the gut microbiota (Dickson et al., 2016; Dickson, 2018; Dickson et al., 2020). In our study, similar changes in composition were also observed in the lung microbiota.

In the normal population, the most dominant phylum in the gut microbiota is *Firmicutes*, followed by *Bacteroidetes*, *Actinobacteria*, and *Proteobacteria* (Bozzi Cionci et al., 2018). In our study, the composition of healthy controls was consistent with the normal population; but in AP patients, *Proteobacteria* significantly increased with disease severity. Previous studies have found *Proteobacteria* overgrowth in patients with AP, particularly SAP (Zhu et al., 2019; Yu et al., 2020; Zhu et al., 2021). In addition, *Proteobacteria* in the lung microbiota are closely associated with inflammatory lung disease and positively related to alveolar TNF-α (Dickson et al., 2016). A higher abundance of *Proteobacteria* was a distinguishing feature of ventilator-associated pneumonia (Fromentin et al., 2021). The enrichment of *Proteobacteria* might be a biomarker of inflammatory status in patients.

In our study, *Enterobacteriaceae* and *Escherichia-Shigella* were dominant in AP-ARDS patients. The overall levels of *Enterobacteriaceae*, and the individual *Enterobacteriaceae* genera, increased significantly in patients with ARDS. *Escherichia-Shigella*, a genus of *Enterobacteriaceae* family, is an opportunistic pathogen and more abundant in the sicker group. Random forest analysis identified *Escherichia-Shigella* as the most significant feature for distinguishing ARDS from non-ARDS. Multiple studies have shown that gut-associated bacteria in the lung microbiota, especially *Enterobacteriaceae*, are more abundant in ARDS patients. The abundance of *Enterobacteriaceae* in the lung microbiota was strongly associated with serum IL-6 level and the development of ARDS (Dickson et al., 2016; Dickson, 2018; Mukherjee and Hanidziar, 2018; Kyo et al., 2019). The composition of *Enterobacteriaceae* in the lung can help to identify ARDS patients (Dickson et al., 2020). The enrichment of gut-associated bacteria could also be a biomarker for ARDS patients (Fromentin et al., 2021). Suppression of the gut microbiota could improve the prognosis of critically ill patients (Silvestri et al., 2012).

At the species level, potentially pathogenic bacteria, including *Klebsiella pneumoniae*, *Prevotella copri*, and *Clostridium ramosum*, increased significantly in AP-ARDS patients. *Klebsiella pneumoniae*, a common pathogen of the *Enterobacteriaceae* family, normally colonizes respiratory tract and gut (Chen et al., 2021; Wolff et al., 2021). Dickson et al. revealed that *Klebsiella pneumoniae* overgrowth in the lung was strongly associated with ARDS (Dickson et al., 2020). In addition, *Klebsiella pneumoniae* infection can influence both the gut microbiome and lung metabolome (Wu et al., 2020; Jiang et al., 2022). After inoculation of mice with *Klebsiella pneumoniae*, the diversity and composition of the gut microbiota changed and contributed to lung microbiota dysbiosis within several hours (Jiang et al., 2022). Therefore, *Klebsiella pneumoniae* in the gut may influence lung inflammation through bacterial translocation. *Prevotella* could increase the host sensitivity to intestinal inflammation (Iljazovic et al., 2021). *Prevotella copri* is the most well-known of the Prevotella genus and is positively correlated with many inflammatory diseases, such as rheumatoid arthritis and ankylosing spondylitis (Tett et al., 2021). Transplantation of *Prevotella copri* induces dysbiosis of inflammatory and immune functions and can induce arthritis in mice (Maeda et al., 2016; Qian et al., 2022). Although less well studied, *Clostridium ramosum* has been proven to be positively correlated with Covid-19 disease severity, as well as infection and bacteremia (Zuo et al., 2020).

In our study, the levels of probiotics, such as *Bifidobacterium* and *Bifidobacterium longum*, decreased in patients with ARDS. *Bifidobacterium longum* was negatively correlated with organ failure and disease severity scores. As a beneficial bacterium, *Bifidobacterium* can help maintain gut barrier function, inhibit bacterial translocation, reduce lung inflammation, and therefore improve prognosis (Akshintala et al., 2019; Zhu et al., 2019; Zhu et al., 2021). In the current study, *Bifidobacterium* decreased in both AP patients and mice (Chen et al., 2017; Huang et al., 2017; Zhu et al., 2019; Li et al., 2020). *Bifidobacterium longum* can inhibit viral-induced lung inflammation and injury in mice (Groeger et al., 2020). Supplementation with *Bifidobacterium longum* has shown promising benefits for many diseases, such as irritable bowel syndrome, atopic dermatitis, and obesity (Schellekens et al., 2021; Fang et al., 2022; Sabaté and Iglicki, 2022). Therefore, probiotics have been used in the treatment of AP despite controversy.

Previous studies found the enrichment of gut-associated bacteria in the lung is closely associated with ARDS. However, whether the changes in the gut and lung microbiota are consistent has never been studied. In our study, the variation in gut microbiota in ARDS patients is similar to those seen in lung microbiota in previous studies, which suggests that changes in the lung microbiota might be due to the translocation of the gut microbiota in ARDS patients.

The gut-lung axis is a potential mechanism by which the gut microbiota influences lung inflammation. Gut microbiota can influence local immunity, systemic inflammation, and host immune suppression (Budden et al., 2017; Mukherjee and Hanidziar, 2018; Siwicka-Gieroba and Czarko-Wicha, 2020). Gut microbiota activate immune cells, which can migrate from the gut to the lung and assist in resisting systemic inflammatory disease (He et al., 2017; Mjösberg and Rao, 2018), and release metabolites and endotoxins to influence host immune response (Segain et al., 2000; Artis, 2008; Lin and Zhang, 2017). Additionally, gut microbiota dysbiosis damages the integrity of the intestinal barrier and enables bacterial translocation (Wang et al., 2022). Bacteria in the gut can translocate to the lung through the lymphatic or blood circulation systems and thus mediate lung inflammation (Mukherjee and Hanidziar, 2018). *Enterobacteriaceae*, *Escherichia-Shigella*, and several gut-associated bacteria have been detected in pancreatic fluid which suggested bacteria translocation could occur in AP patients and lead to infected pancreatic necrosis (Li et al., 2013; Hanna et al., 2014; Schmidt et al., 2014). Further studies have revealed that the composition of the lung microbiota can be easily changed, even if the immigration of gut-associated bacteria is transient (Dickson et al., 2016).

Gut microbiota could be transferred to the lung by several possible mechanisms. First, intestinal mucosal permeability may be impaired owing to dysbiosis of the microbiota. In our functional analysis, there was a difference in anaerobic bacterial composition between the AP-ARDS and AP-nonARDS groups. Dysbiosis of anaerobic bacteria is correlated with intestinal epithelial integrity and promotes overgrowth of pathogenic bacteria (Hong et al., 2018; Zhou and Liao, 2021). The proliferation of pathogenic bacteria can consume fatty acids, change intestinal pH, inhibit the growth of probiotics, and damage the gut chemical barrier (Wang et al., 2022). In addition, the overgrowth of pathogens restricts the function of immune cells, such as Tregs, Th2, and B cells; promotes the production of inflammatory factors, such as IL-1β, IL-6, and TNF-α; and thus damages the gut immune barrier (Zhou and Liao, 2021; Wang et al., 2022). As a normal pathogen, *Escherichis-Shigella* is associated with epithelial cell injury and is strongly correlated with AP and ARDS severity (Zhu et al., 2019; Pan et al., 2021). Through reduced butyrate production and increased oxidative stress, *Escherichis-Shigella* could penetrate the intestinal barrier, reach the basolateral layer, and spread rapidly to adjacent cells (Fokam Tagne et al., 2018; Dong et al., 2020). A second possible mechanism may involve the lung microenvironment which is important for bacterial colonization; normally, the alveolar ecosystem is not appropriate for bacterial reproduction (Dickson et al., 2017), however, the lung barrier could be damaged in AP patients. Previous studies have shown that inflammatory factors can migrate from the gut to the lung, recruit neutrophils in the blood, and cause lung inflammation (Mjösberg and Rao, 2018; Zhou and Liao, 2021). The inflammatory cascade amplified the inflammatory response and provided a more favorable inflammatory lung microenvironment for bacterial colonization. In ARDS patients, the influx of nutrient-rich edema and establishment of stark oxygen gradients will damage the local host defenses of the lung and make it easier for bacteria to translocate from the gut to the lung (Dickson, 2018). Therefore, patients may be more sensitive to disruption of the gut microbiota. However, these hypotheses have not been fully confirmed.

The translocation of bacteria from the gut to the lung has important clinical implications. Previous studies have proposed that the lung microbiota from *bronchoalveolar lavage fluid* (*BAL*) could help distinguish ARDS. However, rectal swabs are simpler to collect than BAL, therefore, gut microbiota data are easier to acquire than the lung; making gut microbiota a better prospect. In addition, our study found that the gut microbiota changed before the ARDS diagnosis. Prior to the occurrence of AP-ARDS, the gut microbiota already had characteristics relating to ARDS. Therefore, gut microbiota can be an important predictor of ARDS. Among them, *Proteobacteria*, *Enterobacteriaceae*, and *Escherichia-Shigella* were also found increased in lung microbiota in previous studies. These bacteria may help build prediction models for AP-ARDS that could assist clinicians in decision-making and prevent the occurrence and development of AP-ARDS.

Considering the potential function of microbiota dysbiosis, restoring immune competence and disturbing microbiota is a promising therapy for AP-ARDS (Mukherjee and Hanidziar, 2018). However, the effects of probiotics on patients with AP remain controversial. Some trials have revealed that probiotic supplements may have no benefit in the clinical outcomes of AP patients (Isenmann et al., 2004; Mazaki et al., 2006; Dellinger et al., 2007; de Vries et al., 2007) and probiotic treatment may even worsen the prognosis of patients with AP. Probiotics could cause bacteremia despite the rarity and transfer of antibiotic resistance from probiotics to pathogenic bacteria may worsen infection (Salminen et al., 2002; Cannon et al., 2005; Connolly et al., 2005; Feld et al., 2008). Besselink et al. illustrated that probiotic supplementation increases the occurrence of organ failure and mortality in patients with SAP (Besselink et al., 2008).

According to our study results, this poor response might be related to the overgrowth of pathogens and a disrupted intestinal mucosal barrier. For example, *Klebsiella pneumoniae* infection can inhibit *Bifidobacterium* production (Jiang et al., 2022) and the abundance of *Prevotella* is negatively associated with *Bacteroides* (Tett et al., 2021). Therefore, reducing pathogenic bacteria may promote the growth of probiotics, reduce barrier damage, and thus improve the efficacy of probiotic supplements. Targeted antibiotics are an effective strategy. Germ-free or antibiotic-treated animals are consistently protected from ARDS, and prophylactic administration of antibiotics decreases both mortality and multiple organ dysfunction syndromes, including ARDS (Dickson, 2016; Dickson, 2018). Supplementation with short-chain fatty acids (SCFAs) is another effective treatment option. Studies have found that oral supplementation with SCFAs could decrease susceptibility to bacterial infection, indicating that adjusting the gut microbiota could prevent bacterial pneumonia (Seki et al., 2021). These treatment concepts can be applied for AP patients to prevent ARDS (Siwicka-Gieroba and Czarko-Wicha, 2020). However, considering the potential harm caused by probiotics and antibiotics, targeted therapy should be provided to high-risk ARDS patients. Therefore, the prediction or early recognition of ARDS is essential. Collecting gut microbiota in the early stages of AP could help recognize and diagnose ARDS, and thus guide clinical management.

Gut microbiota could help identify high risk population for developing ARDS. Early identification gives time for appropriate intervention which could help improve prognosis. However, our study has some limitations. First, the specific role of microbiota changes in the disease is unclear. Our study can only provide correlations and suggest that the microbiota might help predict ARDS. However, the mechanism by which microbiota causes pathological conditions remains unknown. Zhang et al. found that different initial sites of infection could influence lung microbiota in patients with septic ARDS. ARDS patients with initial intrapulmonary infection tend to have higher abundance of gut-associated in lung (Zhang et al., 2022). To determine the specific role of gut-lung axis, it is better to make a more nuanced classification in the future. Second, the 16S rRNA sequence analysis could not predict the real composition and function of the microbiota community because it is based on the 16S rRNA sequence library. 16S rRNA analysis cannot completely replace metagenomic analysis but can help guide further studies. Third, the detection time 16S rRNA is long now. For clinical application, quick PCR kit target to specific bacteria is still needed.

In conclusion, this is the first study to report the relationship between gut microbiota and AP-ARDS. Gut microbiota showed a potential predicting ability for AP-ARDS. Dysbiosis of gut microbiota is strongly correlated with AP-ARDS. *Enterobacteriaceae* and *Escherichia-Shigella* are important prediction biomarkers for AP-ARDS. In the future, gut microbiota in early stage of patients with AP may help predict and allow early recognition of AP-ARDS, aid therapy planning, and thus improve patients’ quality of life and reduce morbidity of ARDS in AP patients. Further studies will improve our understanding of the role of microbiota in ARDS.

## Data availability statement

The datasets presented in this study can be found in online repositories. The names of the repository/repositories and accession number(s) can be found below: https://www.ncbi.nlm.nih.gov/, PRJNA893348.

## Ethics statement

The studies involving human participants were reviewed and approved by Ethics Committee of PUMCH (JS1826). The patients/participants provided their written informed consent to participate in this study.

## Author contributions

XH and ZH conceived this study and drafted the manuscript. RZ and WS performed data analysis and reviewed the manuscript. ZH, RZ, WS, LG, ZY, and XS collected rectal swabs and clinical data. SZ revised the manuscript. HS and DW contributed to the study design, managed this study and revised the manuscript. All authors contributed to the article and approved the submitted version.
